# Supplementary material for: MetaRibo-Seq measures translation in microbiomes
Source: Nat Commun. 2020 Jun 29;11:3268. doi: 10.1038/s41467-020-17081-z (PMC7324362; doi:10.1038/s41467-020-17081-z)
Supplement: Supplementary file 10 — Supplementary Data 7 [file 41467_2020_17081_MOESM10_ESM.zip › File2/Confidence_High_Taxonomy/40471_out.krona.html]

Javascript must be enabled to view this page.

members
magnitude
magnitudeUnassigned
count
unassigned
taxon
rank

40471\_out

3

3
superkingdom
2759

4751
3
kingdom

3
subkingdom
451864

phylum
3
4890

147538
subphylum
3

class
1
147545

451870
1
subclass

order
1
34395

43219
family
1

40354
genus
1


SRS1041145\_contig\_number\_32763
856822
1
species

class
2
147550

222543
subclass
1

1028384
order
1

681950
1
family

5455
genus
1


SRS051031\_contig\_number\_contig-100\_186.199142
1173701
species
1

subclass
1
222544

order
1

SRS023914\_contig\_number\_28410
5139
